# Resolution of Metastatic Subungal Eccrine Porocarcinoma Treated with Intralesional Interleukin-2

**DOI:** 10.3390/curroncol28010024

**Published:** 2020-12-30

**Authors:** Ashley Drohan, Jennifer Melvin, Joanne Murphy, Carman Giacomantonio, Lucy Helyer

**Affiliations:** 1 QEII Health Sciences Centre, Division of General Surgery, Dalhousie University, 1276 South Park St., Halifax, NS B3H 2Y9, Canada; 2 Department of Medical Oncology, Dalhousie University, QEII Bethune Building, 1276 South Park St., Halifax, NS B3H 2Y9, Canada; jn546206@dal.ca; 3 Department of Pathology, Dalhousie University, Halifax, NS B3H 4R2, Canada; Joanne.Murphy@nshealth.ca; 4 Department of Surgery, Dalhousie University, Halifax, NS B3H 2Y9, Canada; carman.giacomantonio@nshealth.ca (C.G.); lhelyer@dal.ca (L.H.)

**Keywords:** porocarcinoma, interleukin-2, immunotherapy

## Abstract

Eccrine porocarcinoma is a rare aggressive cutaneous malignancy. Complete surgical excision is the standard of care, although there are high rates of local and distant recurrence. We present a unique case of locally recurrent and metastatic subungal porocarcinoma successfully treated with intralesional interleukin-2.

## 1. Introduction

Eccrine porocarcinoma is a rare cutaneous malignancy derived from the dermal sweat gland and acrosyringium [1]. It is most often diagnosed in the seventh and eighth decades of life, and males and females are affected equally [2]. The most common locations include head and neck, and lower extremity [3,4]. Porocarcinoma is generally regarded as an aggressive neoplasm, with high rates of local recurrence and metastatic disease.

Given the rarity of porocarcinoma, no current guidelines exist regarding the optimal treatment of this aggressive malignancy; however, complete surgical excision with wide margins is the standard of care [1]. Recently, the use of Moh’s micrographic has been supported in patients with intraepidermal spread [5]. In patients with no palpable clinical lymphadenopathy and tumor depth >7 mm, sentinel lymph node biopsy is also recommended, given that 80% of these patients will have occult lymph node metastasis [4].

Although porocarcinoma is perceived as a chemo-resistant disease, there have been reports of successful treatment of metastatic disease with specific regimens including carboplatin and epirubicin [6], and paclitaxel and cetuximab [7] After obtaining fully informed, voluntary consent, we describe a case of the successful treatment of locally recurrent, metastatic porocarcinoma using a novel therapy, intralesional interleukin (IL)-2. The authors have no conflict of interest to declare.

## 2. Case Report

**Figure 1:**
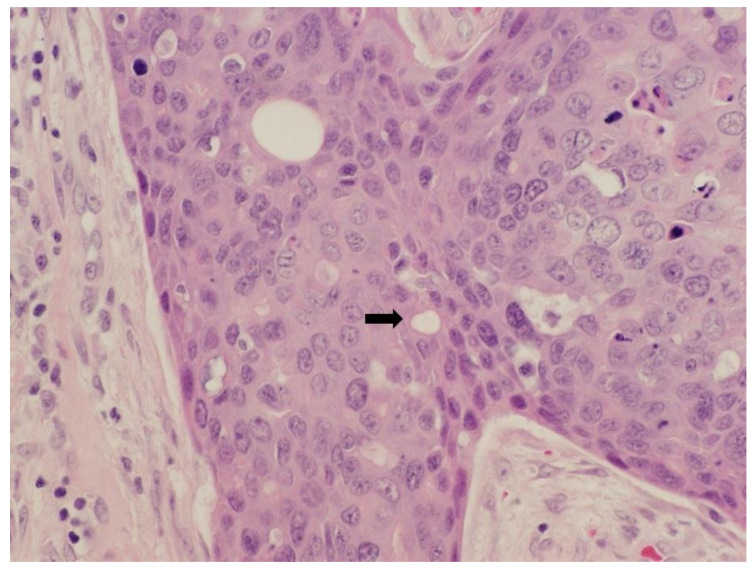
Photomicrograph taken at high power showing invasive porocarcinoma with luminal-like spaces within epithelial nests (40×).

A 56-year-old male presented initially to the plastic surgery clinic with a right thumb lesion that had been present for approximately 2 years, and underwent a wide excision with flap closure. Final pathology revealed invasive porocarcinoma, measuring 1.3 cm, with positive margins and dermal lymphatic involvement, and consisted of nests and cord of atypical, mitotically active epithelial cells involving ulcerated epidermis, dermis, and subcutis without bone involvement. The cells exhibited marked nuclear pleomorphism and focal comedonecrosis. Some epithelial nests contained luminal structures, a distinguishing feature between porocarcinoma and squamous cell carcinoma (Figure 1). Lymphovascular invasion was prominent and extended beyond the main tumor mass to involve the proximal margin.

Within weeks of his original surgery, the patient developed a new nodular recurrence at the suture line and subsequently underwent a partial amputation at the distal interphalangeal joint demonstrating persistent porocarcinoma with lymphatic involvement. At that time, a palpable right axillary node was detected and fine-needle aspiration was positive for malignant cells.

A complete metastatic workup revealed no evidence of distant disease, and he underwent a radical axillary lymph node dissection including nodal levels 1–3; 2/28 nodes were positive with the largest node measuring 3.7 cm. Given the burden of nodal disease, he went on to receive 4800 cGy in 20 fractions of adjuvant axillary external beam radiation.

**Figure 2:**
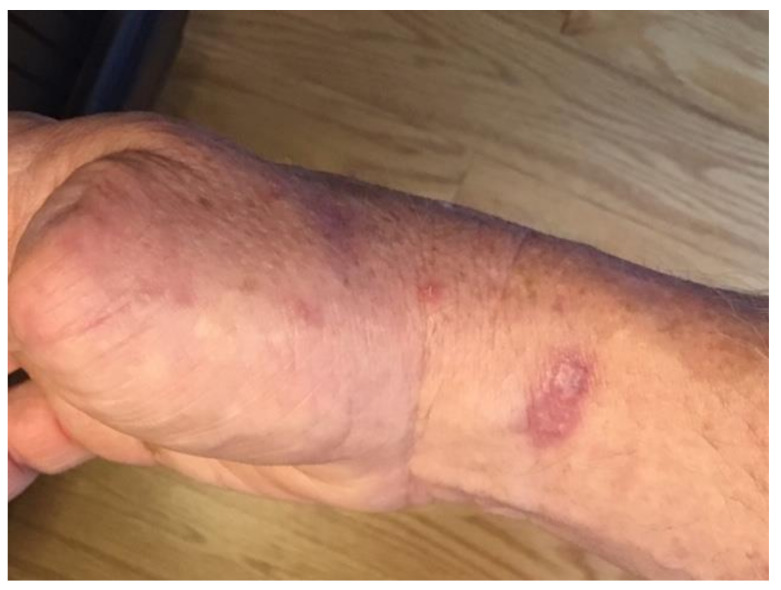
Cutaneous metastasis of invasive porocarcinoma present on right wrist, before interleukin (IL)-2 injection therapy.

Surveillance CT scans at 6 months post lymph node dissection did not reveal any evidence of new metastatic disease; however; within 7 months of surgery, he developed multiple cutaneous lesions with a papular, cystic appearance, located on the right hand and chest (Figure 2).

A biopsy confirmed metastatic porocarcinoma. A re-staging CT did not demonstrate any visceral metastasis. Given the new diagnosis of cutaneous metastasis, he was then treated with palliative intent carboplatin and paclitaxel. After 4 cycles, there was an excellent clinical and radiographic response with almost complete resolution of all lesions and a negative PET CT. However, he developed significant chemotoxicities including neutropenia and peripheral neuropathy, and no further chemotherapy was administered.

Within one month of his last chemotherapy, there was growth of all cutaneous metastasis and development of new lesions. Given that all other treatment modalities had been attempted and failed, the multidisciplinary team decided to proceed with the novel use of intralesional IL-2 in the treatment of this patient’s porocarcinoma. Quoted risks of IL-2 injection include pain, flu-like symptoms, fever, and hypotension. Consent was obtained and each cutaneous met was injected with varying volumes of 500,000 IU/0.1 mL of IL-2, with a total injected volume of 2.4 mL (0.8 mL per vial). Two weeks later, another 5 vials (4 mL) were injected.

Two weeks following the second injection, a dramatic clinical response was appreciated, with decrease in size of all lesions. We continued for a total of 7 injections to all visible lesions every 2–4 weeks. The injected IL-2 volume ranged from 2–4 mL per session. The patient reported minimal side effects, aside from becoming hypotensive after his second treatment, with a systolic blood pressure in the 90s after treatment with 4 mL of IL-2.

**Figure 3:**
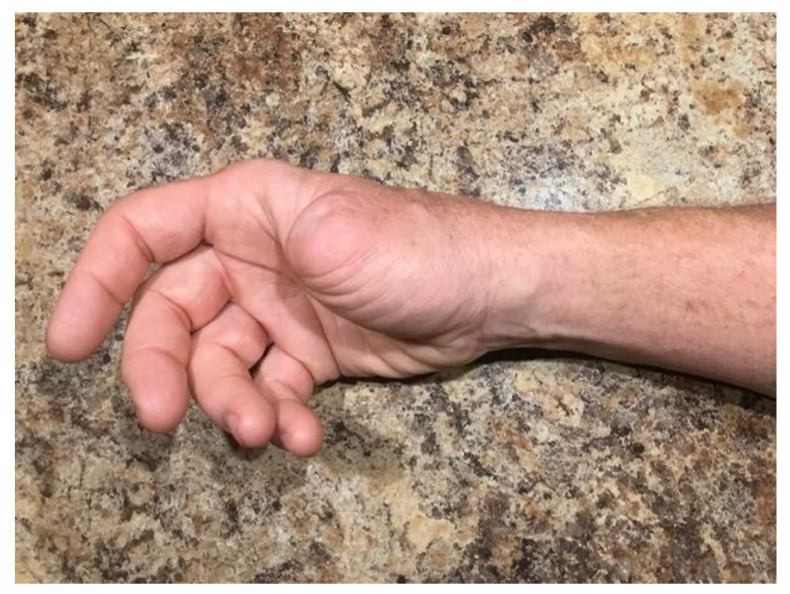
Resolution of right wrist invasive porocarcinoma cutaneous metastasis after completion of IL-2 injection therapy.

Since completing treatment with IL-2, he has undergone a surveillance PET CT scan 6 months after the last dose of IL-2, which revealed no definitive active disease, and biopsies of two separate persistent lesions demonstrated fibrosis without evidence of porocarcinoma. At this point, the patient has completed his intralesional IL-2 treatment, and has no clinical, pathologic, or radiographic evidence of porocarcinoma ten months after his last treatment of IL-2 (Figure 3).

## 3. Discussion

In this case report, we have described the successful clearance of metastatic porocarcinoma, in a patient who had previously been treated with surgical resection, chemotherapy and radiation. The use of intralesional IL-2 for porocarcinoma has, to our knowledge, only been described once before in the literature. In 1992, Dummer et al. described a case of a 74-year-old male with eccrine porocarcinoma of the fifth finger who developed cutaneous metastasis status post-amputation of the fourth and fifth finger and axillary lymphadnopathy [8]. After two treatments with intralesional IL-2 and interferon alpha, there was a resolution of the lesions and the patient remained in remission for at least 20 weeks, when the case report was published. The authors hypothesized that the treatment effect was secondary to the synergy between the two injected cytokines; our case would suggest that this patient may have had a similar response, if treated with IL-2 only.

Other local therapies have been described in the treatment of porocarcinoma. Imiquimod and maxacalcitol, as well as intralesional photodynamic therapy have been used successfully in the treatment of primary porocarcinoma not amenable to surgery [9,10]. Similarly, systemic chemotherapy has achieved remission in patients with dermal and lymphatic porocarcinoma metastasis [11]. However, there is a paucity of data describing the use of local therapy for the treatment of metastatic disease.

IL-2 is a cytokine that plays an important role in inflammation and cellular immunity. It promotes the proliferation and survival of CD4+ and CD8+ T-cells [12]. Given its ability to stimulate T cell proliferation, IL-2 has been used as a cancer immunotherapy since the 1980s, when it was described in the use of metastatic melanoma and renal cell carcinoma [13,14,15]. At present, the use of intralesional IL-2 for metastatic and in-transit melanoma is well supported [16,17]; however its use in other malignancies is not well described.

In addition to demonstrating the successful treatment of a rare, aggressive malignancy, this case also highlights an uncommon presentation of porocarcinoma and illustrates the difficulty around diagnosis. The most common location for porocarcinoma is the head and neck, and the lower extremity [2,4]. In a recent systematic review of 206 cases of porocarcinoma reported between 1963 and 2017, only 3 (1.4%) were located on the hand [4]. Subungual porocarcinoma, as in this case, is even more rare, with only two reports of porocarcinoma located in the nail bed of the hand [18,19]. This highlights the importance for clinicians to maintain a high index of suspicion for malignancy in any patient with a non healing lesion of the subungual area. As our case demonstrates, porocarcinoma is an aggressive malignancy with a tendency to recur locally and metastasize. Early diagnosis may improve outcomes.

In summary, we present a case of a 56-year-old male with locally recurrent subungual porocarcinoma of the right thumb with axillary and cutaneous metastatic disease. He was successfully treated with thumb amputation, radical axillary lymphadenectomy, and axillary external beam radiation for local control and intralesional IL-2 for the control of metastatic cutaneous lesions. Ten months after his last IL-2 therapy, he has no clinical, pathologic, or radiographic evidence of disease, and his injections were well tolerated. We propose that the use of intralesional IL-2 in the treatment of metastatic porocarcinoma warrants further investigation and represents a potential disease altering treatment for a rare and aggressive malignancy.

## References

[1] Rongioletti F., Mărgăritescu I., Smoller B.R. (2015). Rare Malignant Skin Tumors.

[2] Salih A.M., Kakamad F.H., Essa R.A., Rauf G.M., Masrur S.A., Shvan H.M., Rawezh Q.S., Hunar A.H., Dahat A.H., Othman S. (2017). Porocarcinoma: A systematic review of literature with a single case report. Int. J. Surg. Case Rep..

[3] Riera-Leal L., Guevara-Gutiérrez E., Barrientos-García J.G., Madrigal-Kasem R., Briseño-Rodríguez G., Tlacuilo-Parra A. (2015). Eccrine porocarcinoma: Epidemiologic and histopathologic characteristics. Int. J. Dermatol..

[4] Nazemi A., Higgins S., Swift R., In G., Miller K., Wysong A. (2018). Eccrine Porocarcinoma. Dermatol. Surg..

[5] Tolkachjov S.N., Hocker T.L., Camilleri M.J., Baum C.L. (2016). Treatment of Porocarcinoma With Mohs Micrographic Surgery. Dermatol. Surg..

[6] Fukuda K., Tabolli S., Fukuyama M., Kakuta R., Sato M., Nakamura Y., Tanese K., Masugi Y., Amagai M. (2015). Metastatic eccrine porocarcinoma successfully treated with carboplatin and epirubicin chemotherapy. J. Dermatol..

[7] Godillot C., Boulinguez S., Riffaud L., Sibaud V., Chira C., Tournier E., Paul C., Meyer N. (2018). Complete response of a metastatic porocarcinoma treated with paclitaxel, cetuximab and radiotherapy. Eur. J. Cancer.

[8] Dummer R., Becker J.C., Boser B., Hartmann A.A., Burg G. (1992). Successful Therapy of Metastatic Eccrine Poroma Using Perilesional Interferon Alfa and Interleukin 2. Arch. Dermatol..

[9] Nagasawa T., Hirata A., Niiyama S., Enomoto Y., Fukuda H. (2019). Successful treatment of porocarcinoma with maxacalcitol and imiquimod. Dermatol. Ther..

[10] Torchia D., Amorosi A., Cappugi P. (2015). Intralesional Photodynamic Therapy for Eccrine Porocarcinoma. Dermatol. Surg..

[11] Imafuku K., Hata H., Kitamura S., Iwata H., Shimizu H. (2015). In-transit metastasis of advanced eccrine porocarcinoma. Int. J. Dermatol..

[12] A Rosalia R., Arenas-Ramirez N., Bouchaud G., Raeber M.E., Boyman O. (2014). Use of enhanced interleukin-2 formulations for improved immunotherapy against cancer. Curr. Opin. Chem. Biol..

[13] Klapper J.A., Downey S.G., Smith F.O., Yang J.C.-H., Hughes M.S., Kammula U.S., Sherry R.M., Royal R.E., Steinberg S.M., Rosenberg S. (2008). High-dose interleukin-2 for the treatment of metastatic renal cell carcinoma. Cancer.

[14] Atkins M.B., Lotze M.T., Dutcher J.P., Fisher R.I., Weiss G., Margolin K., Abrams J., Sznol M., Parkinson D., Hawkins M. (1999). High-Dose Recombinant Interleukin 2 Therapy for Patients with Metastatic Melanoma: Analysis of 270 Patients Treated Between 1985 and 1993. J. Clin. Oncol..

[15] Smith F.O., Downey S.G., Klapper J.A., Yang J.C., Sherry R.M., Royal R.E., Kammula U.S., Hughes M.S., Restifo N.P., Levy C.L. (2008). Treatment of Metastatic Melanoma Using Interleukin-2 Alone or in Conjunction with Vaccines. Clin. Cancer Res..

[16] Hassan S., Petrella T.M., Zhang T., Kamel-Reid S., Nordio F., Baccarelli A., Sade S., Naert K., Al Habeeb A., Ghazarian D. (2014). Pathologic Complete Response to Intralesional Interleukin-2 Therapy Associated with Improved Survival in Melanoma Patients with In-Transit Disease. Ann. Surg. Oncol..

[17] Nouri N., Garbe C. (2016). Intralesional immunotherapy as a strategy to treat melanoma. Expert Opin. Biol. Ther..

[18] Palleschi G.M., Dragoni F., Urso C. (2017). Subungual Eccrine Porocarcinoma. Dermatol. Surg..

[19] Bhari N., Arava S.K., Jangid B.L., Srivastava A., Sharma V.K. (2017). A fleshy growth below the nail plate in an elderly man. Int. J. Dermatol..

